# Effects of fermented red bean extract on nephropathy in streptozocin-induced diabetic rats

**DOI:** 10.29219/fnr.v64.4272

**Published:** 2020-12-22

**Authors:** Kung-Chi Chan, Kar-Eng Kok, Keh-Feng Huang, Yao-Lin Weng, Yun-Chin Chung

**Affiliations:** 1 Department of Food and Nutrition, Providence University, Taichung, Republic of China (Taiwan); 2 Department of Applied Chemistry, Providence University, Taichung, Republic of China (Taiwan)

**Keywords:** advanced-glycation end-products, C-reactive protein, 24-hour creatinine clearance, diabetic rats, natto-red bean extract

## Abstract

**Background:**

The antioxidant effects of *Bacillus subtilis*-fermented red bean (natto-red bean) extract (NRBE) in young (6 weeks old) Sprague–Dawley rats and aged (12 months old) mice had been reported previously.

**Objective:**

To evaluate the antioxidant and anti-inflammatory effects of NRBE in the kidneys of streptozocin-induced diabetic rats.

**Design:**

Normal control rats and diabetic rats were orally gavaged with saline and low-dose NRBE (100 mg/kg body weight [BW]), medium-dose NRBE (200 mg/kg BW), and high-dose NRBE (500 mg/kg BW), for 12 weeks and then sacrificed. Concentration of fasting glucose, adiponectin, renal function markers, antioxidative markers, and pro-inflammatory markers were measured.

**Results:**

Oral administration of 50% ethanolic extract of NRBE with a dosage of 100 mg/kg BW, 200 mg/kg BW, or 500 mg/kg BW could improve the symptoms of kidney enlargement and renal function. Supplementation of NRBE can effectively inhibit the formation of renal reactive oxygen species and advanced-glycation end-products and increase renal glutathione content and serum adiponectin. A low dose of NRBE (100 mg/kg BW) decreased fasting blood sugar and renal interleukin (IL)-6 expression. Serum C-reactive protein, renal tumor necrosis factor-α, and monocyte chemoattractant protein-1 concentrations were decreased, and renal superoxide dismutase activity was increased in the medium-dose NRBE group. Twenty-four hour creatinine clearance and urinary albumin excretion also improved by medium-dose NRBE supplementation. In NRBE, total phenols and flavonoids were 6.3 mg gallic acid equivalent/g and 12.02 mg rutin equivalent/g, respectively, and kampherol was the major active antioxidant compound.

**Conclusion:**

This study demonstrated that appropriate amount of NRBE, 200 mg/kg BW in rats, could prevent diabetic nephropathy by improving antioxidant status and inhibiting inflammation in renal tissue.

## Popular scientific summary

- Natto-red bean extract (NRBE) is a functional ingredient with antioxidant and anti-inflammatory properties.
- NRBE decreases renal ROS and AGE formation, and increases renal glutathione and serum adiponectin in diabetic rats.
- NRBE decreases serum CRP, renal TNF-a and MCP-1, and increases renal SOD activity.
- NRBE improves renal function of diabetic rats, as judged by 24 hrs CCr and urinary albumin excretion (UAE) parameters.

The global prevalence of diabetes among adults older than 18 years has risen from 4.7% in 1980 to 8.5% in 2014, and an estimated 1.6 million deaths were directly caused by diabetes in 2015, according to a World Health Report on November 15, 2017. WHO projected that diabetes was the eighth leading cause of death in 2011. It is estimated that there will be 693 million people living with diabetes mellitus (DM) in the world by the year 2045 (1). Type 2 diabetes mellitus (T2DM) results from the body’s ineffective use of insulin, and most people with diabetes around the world have T2DM. In 2017, it was reported that chronic kidney failure was the first leading major disease at outpatient clinics in Taiwan (2). End-stage kidney failure always results from chronic kidney disease (CKD). Diabetic kidney disease develops in approximately 40% of diabetic patients and is the leading cause of CKD worldwide (3).

Oxidative damage induced by hyperglycemia is one of the major causes of diabetic nephropathy (DN), and this disease includes kidney failure and high risk for macrovascular diseases, which may lead to death (4). When hyperglycemia status is persistent, a significant amount of reactive oxygen species (ROS) will be generated by mitochondria in vascular endothelial cells, which leads to high oxidative stress in cells, induces inflammation, increases advanced-glycation end-products (AGEs), and results in serious micro- and macrovascular complications (5, 6). Therefore, improving DN by preventing oxidative stress and subsequent chronic inflammation induced by hyperglycemia is of major importance.

Hyperglycemia-induced oxidative stress is a key component that contributes to the formation of DN (7). Moreover, diabetic nephrological progression is accompanied by the excessive production of proinflammatory cytokines such as interleukin (IL)-6 and tumor necrosis factor (TNF-α), which exacerbate systemic inflammatory stress and diabetic deterioration (8). Therefore, any agent with antioxidative and/or anti-inflammatory activities may potentially delay the development of DN.

In Asia, soybean substrates are commonly used in the production of microbial-fermented foods, such as miso fermented by *Aspergillus oryzae*, tempeh fermented by *Rhizopus oligosporus*, and natto fermented by *Bacillus subtilis* (9). Red beans are seldom fermented. In Chinese folk medication, red beans are commonly used for the treatment of constipation, anemia, and edema antidiabetic agent (10).

Natto is a traditional Japanese fermented bean product. The natto kinase in natto products provides the fibrinolytic activity of natto, which is effective in preventing thrombosis and other related diseases (11). Natto kinase proceeds three stages of anti-thrombosis function: converting plasminogen into plasmin, activating tissue plasminogen activator, which also causes the transformation of plasminogen into plasmin, and degradation of fibrin (12). In our previous studies, red beans replaced soybeans in fermentation with *B. subtilis* (the product was called ‘natto-red beans’ extract or NRBE), which exerted fibrinolytic activity as well (12). The fibrinolytic enzyme, a subtilisin-like serine protease, was purified from NRBE (12). In addition, NRBE showed excellent antioxidant activities (13). Research showed that 50% ethanolic extract of *B. subtilis*-fermented red bean could enhance the antioxidant capacity of rats (14). The 50% ethanolic extract of NRBE could enhance the antioxidant levels in liver tissue and completely recover the aging-related change of malondialdehyde (MDA) levels and plasma antioxidant status (14, 15).

In the present study, we propose the hypothesis that NRBE could prevent DN by improving antioxidant status and inhibiting inflammation in renal tissue.

## Methods

### Chemicals

Bovine serum albumin, streptozocin, triethanolamine, and α-chloralose were purchased from Sigma-Aldrich (St. Louis, MO, USA). AssayMax Rat adiponectin enzyme-linked immunosorbent assay (ELISA) kit was purchased from Assaypro (St. Charles, MO, USA). The creatinine reagent set and urea nitrogen reagent set were purchased from Teco Diagnostics (Anaheim, CA, USA). The rat microalbumin ELISA kit was obtained from Kamiya Biomedical Company (Gateway Drive, Seattle, USA).

Rat C-reactive protein (CRP) ELISA kit, rat IL-6 Legend Max^TM^ ELISA kit, and rat TNF-α Legend Max^TM^ ELISA kit were obtained from BioLegend (San Diego, CA, USA). Rat monocyte chemoattractant protein-1 (MCP-1) ELISA kit was obtained from RayBiotech (Norccoss, GA, USA). Rat CRP ELISA kit was obtained from Immunology Consultants Laboratory, Inc. (Portland, OR, USA). OxiSelect^TM^ *In Vitro* ROS/RNA assay kit and OxiSelect^TM^ advanced glycation end competitive ELISA kit were obtained from Cell Biolabs (San Diego, CA, USA). Superoxide dismutase (SOD) assay kit, glutathione (GSH) assay kit, and GSH peroxidase (GPx) assay kit were obtained from Cayman Chemical Company (Ann Arbor, MI, USA). Bio-Rad Protein Assay Kit was obtained from Bio-Rad (Hercules, CA, USA).

### Preparation of NRBE

*B. subtilis* (BCRC 14716) was purchased from the Bioresource Collection and Research Center (Food Industry Research and Development Institution, Hsinchu, Taiwan). Starter was prepared according to Jhan et al. (16).

Red bean (*Vigna angularis*, Kaohsiung #8) was obtained from the Kaohsiung District Agriculture Improvement Station, Kaohsiung, Taiwan. The bean (500 g) was soaked with 500 mL of dd H_2_O for 8 h in a 28 cm (L) × 20 cm (W) × 5.5 cm (H) stainless-steel container covered with an aluminum foil. The solid-state fermentation was initiated by adding 100 mL of sterile, 15% glucose, and 50 mL of inoculum (9.5 log Colony Forming Unit (CFU)/mL) into steamed (121°C for 1 h) and cooled red beans and then incubating at 37°C for 24 h. Subsequently, 100 g of *B. subtilis*-fermented red beans (NRBE) was extracted with 2 L of 50% ethanol solution at 25°C with stirring for 3 h. The mixture was centrifuged at 11,000 × *g* for 10 min, and the suspension was collected and then dried by a freeze dryer. The dry matters of extracts were sealed in plastic bags and stored at -20°C until use.

### Animal treatment

Male healthy Wistar rats (4 weeks old) were purchased from BioLASCO Taiwan Co., Ltd (Taipei, Taiwan). The rats were individually housed in suspended stainless-steel wire-bottom cages (cage size: 47.3 × 25.5 × 21.5 cm) in an environmentally controlled room (22 ± 2°C, 50 ± 5% relative humidity) under a 12-h night and dark cycle with free access to standard rodent chew (Fu-So pellet chow Taichung, Taiwan) and water. All animals received humane care according to the guideline of the Guidebook for the Care and Use of Laboratory Animals (17). The study protocol was approved by the animal research ethics committee.

The rats were weighed daily to assess health status. Rats were divided into five groups (*n* = 8) when their body weight (BW) reached approximately 200 g. Except for the normal control (NC) group, all rats were fasted for 8 h before intraperitoneal injection of streptozocin (45 mg/kg BW) dissolved in 0.1 M citric acid buffer (pH 4.2–4.5) for 3 continuous days to induce diabetes. The feeding of NRBE (0, 100, 200, and 500 mg/kg/day) was achieved by daily oral gavage for 12 consecutive weeks.

### Tissue sampling and preparation

At the end of the experiment, the animals were anesthetized with 5 mL/kg BW, 0.02% α-chloralose, 0.02% sodium tetraborate decahydrate, and 0.1% urethane. The blood was collected into sterile tubes. The serum was prepared by centrifugation at 805 × *g* for 10 min at 4°C and stored at -20°C for further analyses. Tissue samples including heart, liver, and brain were stored at -80°C. The tissue cytosols were the clear supernatants prepared by homogenizing tissues with 50 mM sodium phosphate buffer (pH 7.4) containing 2% triton X-100 followed by centrifugation at 16,543 × *g* for 10 min at 4°C. The serum samples were analyzed for the concentration of triglyceride and total cholesterol, as well as the total antioxidant status. In addition, the renal tissue homogenates were analyzed for MDA and GSH levels as well as the activities of antioxidant enzymes. This study protocol was approved by the Animal Research Ethics Committee.

### Biochemical analysis of blood

Blood was collected by tail vein sampling and centrifuged at 200 × *g* for 15 min. The sugar concentration was measured by Glucose & Lactose analyzer (YSI 2300, Giangarlo Scientific Co., PA, USA).

The concentrations of adiponectin, blood urea nitrogen (BUN), and creatinine were measured following instructions of the AssayMax Rat adiponectin ELISA kit, urea nitrogen reagent set, and creatinine reagent set, respectively.

### Biochemical analysis of urine

Urinary creatinine levels were measured using the creatinine reagent set. The creatinine clearance (Ccr), an effective index for expressing the glomerular filtration rate (GFR), was calculated using the following equation:

$$Ccr\;(mL/\text{​}min)=\frac{Urine\;creatinine}{Serum\;creatinine}\times \frac{24\;h\;urine\;volume\;(mL)}{1,440\;\min}$$

Urinary albumin excretion (UAE) concentration was determined using the rat microalbumin ELISA kit.

### Antioxidant activity of renal tissue

ROS concentration was determined using an *in vitro* ROS/RNA assay, and the level of ROS was calculated as the relative fluorescence of treated versus untreated samples. GSH was measured using the GSH assay kit, and the concentration of GSH was expressed as μM/mg protein in renal tissue.

GPx and SOD activities were assayed using the GPx assay kit and SOD assay kit, respectively, and expressed as nmol/min/mg protein and U/mg protein in renal tissue, respectively.

### Inflammatory analysis of renal tissue

Inflammation-related cytokines, including both IL-6 and TNF-α, were measured using the rat IL-6 Legend Max^TM^ ELISA kit and rat TNF-α Legend Max^TM^ ELISA kit, respectively, and expressed as pg/mg protein in renal tissue.

Concentrations of MCP-1 and CRP were determined using a rat MCP-1 ELISA kit and rat CRP ELISA kit, respectively, and expressed as pg/mg protein and mg/mL, respectively.

The concentration of AGEs was determined using the OxiSelect^TM^ advanced-glycation end-competitive ELISA kit and expressed as ng/mg protein.

### Protein concentration analysis

Protein concentration was determined using the Bio-Rad protein assay kit.

### Identification of active compound

NRBE (500 g) was mixed with distilled water (1,000 mL) and heated to dissolve. Equal volume (1,000 mL) of distilled water and ethyl acetate (EtOAc) was mixed, and 1,000 mL of water-saturated EtOAc was used for partition extraction. The NRBE solution was extracted twice with water-saturated EtOAc, and the EtOAc fraction was concentrated and freeze-dried.

The powders (21.68 g) and silica gel (120 g) were mixed and packed into a 60 cm (H) × 4 cm (D) column with N-hexane flowing. Chromatography was performed with N-hexane:EtOAc in the order of 1:0, 19:1, 9:1, 7:3, 5:5, and 3:7. Each 100 mL elute was collected, concentrated, and freeze-dried for further assay of antioxidant activities, α-iphenyl-β-picryl-hydrazyl (DPPH) scavenge, and lipid peroxidation inhibition. Fractions of 7, 8, 23, 39, 40, 42, 43, 44, 45, and 46, which showed high activity either on DPPH scavenge or lipid peroxidation inhibition, were combined and recrystallized, and then identified by 13C NMR.

### Statistical analysis

All measurements were conducted in triplicate. Data are expressed as mean ± standard deviation. Mean values were compared by Student’s *t* test or analysis of variance followed by Fisher’s least significant difference using SPSS 20.0 software (SPSS Inc., Chicago, IL, USA). A significance level of 5% was adopted for all comparisons.

## Results

### Body weight and food and water intake

There was no significant difference in initial BW between rats assigned as the diabetic control (DC) group and the NC group (*P* > 0.05); however, the DC group showed higher food and water intake and lower food efficiency ratio, final BW and daily weight gain compared with the NC group (Table 1; *P* < 0.05). Regarding the parameters mentioned earlier, the DC group did not show a significant difference from any treatment group (*P* > 0.05).

**Table 1:** The effect of fermented red bean extract (NRBE) on food intake, water intake, body weight, and relative weight of liver and kidneys in diabetic rats.

Group	NC	DC	L	M	H
Food intake (g/day)	33.0 ± 2.5	76.3 ± 3.9^*a^	71.6 ± 4.2^a^	69.5 ± 4.8^a^	70.4 ± 5.6^a^
Water intake (mL/day)	54.3 ± 3.6	347.6 ± 11.8^*a^	320.2 ± 11.4^a^	329.5 ± 13.2^a^	308.4 ± 9.2^a^
Initial body weight (g)	211.4 ± 5.5	208.6 ± 3.1	201.9 ± 5.3	206.8 ± 1.9	209.4 ± 3.3
Final body weight (g)	574.6 ± 43.3	359.9 ± 9.0^*a^	363.9 ± 9.74^a^	387.1 ± 19.2^a^	405.1 ± 13.4^a^
Daily weight gain (g)	3.6 ± 0.0	1.2 ± 0.1^*a^	1.5 ± 0.1^a^	1.9 ± 0.2^a^	1.6 ± 0.1^a^
Liver (g/100g BW)	2.27 ± 0.02	4.50 ± 0.10^*a^	4.05 ± 0.06^b^	3.88 ± 0.08^b^	3.89 ± 0.02^b^
Kidneys (g/100g BW)	0.51 ± 0.01	1.23 ± 0.26^*a^	1.05 ± 0.02^b^	1.06 ± 0.12^b^	1.03 ± 0.25^b^

^1^Values are mean ± SEM, 8 rats per group.

^2^NC: normal control, DC: diabetic control, L: diabetes with low dose of NRBE (100 mg/kg BW), M: diabetes with medium dose of NRBE (200 mg/kg BW), H: diabetes with high dose of NRBE (500 mg/kg BW).

* Significantly different with NC at *p* < 0.05.

a~b Means not sharing a common superscript in the same row are significantly different at *p* < 0.05.

Compared with the NC group, the DC group showed a significantly higher relative weight of liver and kidney (*P* < 0.05). Oral administration of NRBE significantly reduced the relative weight of the liver and kidney in diabetic rats (*P* < 0.05), regardless of the tested dosages.

### Effects of NRBE on blood sugar and adiponectin of diabetic rats

The DC group and all NRBE-treated groups showed significantly higher fasting blood glucose levels than the NC group (*P* < 0.05), indicating that the DC group and all NRBE-treated groups were induced to diabetes by streptozocin before experimental treatments (Table 2).

**Table 2:** Effect of fermented red bean extract (NRBE) on fasting glucose and adiponectin concentration in diabetic rats.

NRBE treatment	Measurement	NC	DC	L	M	H
Before	Fasting glucose (mg/dL)	88.0 ± 17.9	383.1 ± 11.8^*a^	385.8 ± 26.4^a^	385.5 ± 74.8^a^	387.3 ± 24.7^a^
After	Fasting glucose (mg/dL)	91.3 ± 3.21	489.6 ± 19.2^*a^	444.5 ± 20.6^b^	456.4 ± 12.0^ab^	470.1 ± 6.5^ab^
	Adiponectin (μg/dL)	6.6 ± 0.2	2.0 ± 0.3^*a^	3.6 ± 0.4 ^b^	4.4 ± 0.3^b^	3.9 ± 0.3^b^

^1^Values are mean ± SEM, 8 rats per group.

^2^NC: normal control, DC: diabetic control, L: diabetes with low dose of NRBE (100 mg/kg BW), M: diabetes with medium dose of NRBE (200 mg/kg BW), H: diabetes with high dose of NRBE (500 mg/kg BW).

* Significantly different with NC at *p* < 0.05.

a~b Means not sharing a common superscript in the same row are significantly different at *p* < 0.05.

Compared with DC group, diabetic rats fed with medium-dose (200 mg/kg BW) or high-dose NRBE (500 mg/kg BW) did not show a significant change in blood sugar (*P* > 0.05), but a low dose of NRBE (100 mg/kg BW) could reduce the blood sugar level in diabetic rats (Table 2). Table 2 also shows that all blood samples of NRBE-treated rats contained a higher adiponectin concentration compared with those of the DC group.

### Effects of NRBE on renal functions of diabetic rats

Table 3 shows that BUN, urine creatinine, UAE, and Ccr were significantly higher in the DC group (*P* < 0.05). NRBE treatments reduced the change of Ccr and UAE induced by streptozocin (*P* < 0.05), but not that of either BUN or urine creatinine (*P* > 0.05). Medium-dose NRBE had the greatest effect on Ccr level.

**Table 3:** Effect of fermented red bean extract (NRBE) on blood urea nitrogen (BUN), serum creatinine, urine creatinine, 24-h urine creatinine clearance rate (Ccr), and urinary albumin excretion (UAE) in diabetic rats.

Group	NC	DC	L	M	H
BUN (mg/dL)	19.25 ± 0.81	43.41 ± 1.41^*a^	42.65 ± 2.74^a^	39.77 ± 4.65^a^	40.81 ± 2.02^a^
Serum creatinine (mg/mL)	1.79 ± 0.12	2.26 ± 0.13	2.15 ± 0.14	2.42 ± 0.28	1.94 ± 0.08
Urine creatinine (mg/24 hr)	61.07 ± 5.91	10.04 ± 0.25^*a^	10.89 ± 0.16^a^	11.50 ± 0.21^ab^	10.77 ± 0.07^a^
24-h Ccr (mL/min)	0.75 ± 0.19	1.11 ± 0.29^*a^	0.96 ± 0.24^b^	0.77 ± 0.27^c^	1.06 ± 0.16^ab^
UAE (mg/min)	6.80 ± 1.8	53.85 ± 2.05^*a^	28.44 ± 2.80^b^	23.90 ± 3.28^b^	30.47 ±1.12^b^

^1^Values are mean ± SEM, 8 rats per group.

^2^NC: normal control, DC: diabetic control, L: diabetes with low dose of NRBE (100 mg/kg BW), M: diabetes with medium dose of NRBE (200 mg/kg BW), H: diabetes with high dose of NRBE (500 mg/kg BW).

* Significantly different with NC at *p* < 0.05.

a~b Means not sharing a common superscript in the same row are significantly different at *p* < 0.05.

### Effects of NRBE on antioxidant acidity of diabetic rats

Compared with NC rats, DC rats showed significantly higher ROS, AGEs, and GPx levels and lower GSH and SOD levels in the kidneys (Table 4). Oral administration of NRBE could significantly reverse the change in antioxidant status caused by streptozocin (*P* < 0.05). There was no dosage effect on the tested dose, except the medium dose of NRBE that showed the greatest improvement in SOD activity.

**Table 4:** Effect of fermented red bean extract (NRBE) on reactive oxygen species (ROS), glutathione (GSH), advanced glycation end-product (AGEs), and activities of glutathione peroxidase (GPx) and superoxide dismutase (SOD) in kidneys of diabetic rats.

Group	NC	DC	L	M	H
ROS (% of control)	100 ± 0.0	152.5 ± 14.3^*a^	102.4 ± 8.7^b^	76.8 ± 10.4^b^	85.3 ± 10.46^b^
GSH (μM/mg protein)	43.3 ± 4.7	15.0 ± 2.6^*a^	25.4 ± 2.2^b^	24.1 ± 2.3^b^	26.0 ± 1.3^b^
AGEs (μg/mg protein)	347.5 ± 55.2	568.9 ± 32.0^*a^	332.3 ± 36.1^b^	287.2 ± 28.7^b^	327.5 ± 44.6^b^
GPx (nmol/min/mg protein)	0.19 ± 0.1	1.5 ± 0.2^*a^	0.4 ± 0.1^b^	0.31 ± 0.1^b^	0.09 ± 0.0^b^
SOD (U/mg protein)	4.7 ± 0.7	2.6 ± 0.4^*a^	4.0 ± 0.4^a^	4.5 ± 0.9^b^	2.3 ± 0.3^a^

^1^Values are mean ± SEM, 8 rats per group.

^2^NC: normal control, DC: diabetic control, L: diabetes with low dose of NRBE (100 mg/kg BW), M: diabetes with medium dose of NRBE (200 mg/kg BW), H: diabetes with high dose of NRBE (500 mg/kg BW).

* Significantly different with NC at *p* < 0.05.

a~b Means not sharing a common superscript in the same row are significantly different at *p* < 0.05.

### Effects of NRBE on inflammation of diabetic rats

Table 5 shows that the DC group had a significantly higher CRP level than the NC group, indicating that the DC group suffered from inflammation (*P* < 0.05). Both high and low doses of NRBE could not prevent inflammation of the kidneys caused by high glucose levels. Medium-dose NRBE efficiently improved inflammation by down-regulating IL-6, TNF-α, and MCP-1 levels.

**Table 5:** Effect of fermented red bean extract (NRBE) on serum C-reactive protein, interleukin-6 (IL-6), tumor necrosis factor (TNF-α), and monocyte chemoattractant protein–1 (MCP-1) in kidneys of diabetic rats.

Group	NC	DC	L	M	H
CRP (ng/mL)	30.6 ± 1.6	42.0 ± 0.9^*a^	39.5 ± 4.2^ab^	32.7 ± 3.1^b^	34.7 ± 2.0^a^
IL-6 (pg/mg protein)	11.16 ± 1.14	27.94 ± 3.65^*a^	14.40 ± 2.84^b^	20.43 ± 8.22^a^	22.73 ± 2.79^a^
TNF-α (pg/mg protein)	3.43 ± 0.12	6.41 ± 0.59^*a^	5.85 ± 0.65^a^	3.93 ± 0.56^b^	5.76 ± 0.91^a^
MCP-1 (μg/mg protein)	1.63 ± 0.28	4.22 ± 1.06^*a^	2.79 ± 0.43^a^	1.47 ± 0.28^b^	2.76 ± 0.61^a^

^1^Values are mean ± SEM, 8 rats per group.

^2^NC: normal control, DC: diabetic control, L: diabetes with low dose of NRBE (100 mg/kg BW), M: diabetes with medium dose of NRBE (200 mg/kg BW), H: diabetes with high dose of NRBE (500 mg/kg BW).

* Significantly different with NC at *p* < 0.05.

a~b Means not sharing a common superscript in the same row are significantly different at *p* < 0.05.

### Active compound

Total phenols and flavonoids were 6.3 mg gallic acid equivalent/g and 12.02 mg rutin equivalent/g in NRBE, respectively (data not shown). The half-inhibition concentration of NRBE and EtOAc fraction (IC_50_) on DPPH scavenge activity were 3.4 and 1.1 mg/mL, respectively (data not shown). After chromatography, fractions of 7, 8, 23, 39, 40, 42, 43, 44, 45, and 46, which showed high activity either on DPPH scavenge or lipid peroxidation inhibition, were combined (Supplementary Table S1), recrystallized, and subjected for 13C NMR. Result of 13C NMR revealed that kampherol was the major antioxidant compound.

## Discussion

### NRBE improved the renal function of diabetic rats

BUN and Cr, 24-h Ccr, and UAE are commonly used markers to indicate renal function. In clinical tests, it is generally recognized that BUN and Cr are insensitive markers for assessing renal function. However, UAE is an important marker in the early stage of renal diseases (18). In this study, there were significant differences between the DC group and NC group in BUN, 24-h Ccr, and UAE levels, indicating that renal damage was induced successfully by streptozocin injection in the DC group. When compared with the DC group, our data revealed that 24-h Ccr and UAE decreased significantly by supplementing NRBE in diabetic rats. We believe that the present study is the first animal study to investigate the anti-nephropathy effect of NRBE under diabetic conditions.

The 24-h urine collection method or one of the GFR estimation formulas can more accurately identify the decline in kidney function. It is known that renal damage will decrease the urine Ccr rate; however, in our study, we observed that 24-h Ccr was higher in all streptozocin-induced diabetic groups than in the NC group, and similar results have been obtained by several other studies (19, 20). Sharma and Sharma (21) proposed that kidneys are under hyperfiltration status at early stage of renal damage, and consequently, the 24-h Ccr rate is increased at first and then declines gradually at the end stage of CKD. The high Ccr levels in all streptozocin-induced groups also confirmed that all diabetic rats suffered from early renal lesion in this relatively short-term animal study. Hyperfiltration of the glomerulus may be caused by the dilation of the glomerular efferent arteriole and simultaneous constriction of the afferent arteriole, resulting in elevated glomerular pressure (22). An increased intraglomerular pressure may contribute to the hyperfiltration status of the kidneys (23). It is possible that at the early stage of CKD, microvascular lesions of the nephron may increase the filtrating pressure of the glomerulus, leading to an increase in renal blood flow rate and then increased GFR. Oral administration of NRBE decreased the 24-h Ccr, indicating an improvement in early renal lesion, especially in those rats supplemented with medium-dose NRBE (200 mg/kg BW), which can correct the 24-h Ccr to nearly normal levels. Therefore, NRBE supplementation seems to be effective for preventing microvascular lesions of the nephron under diabetic conditions.

Hyperglycemia-induced oxidative damage may lead to the thickening of glomerular basement membrane, sclerosis of glomerulus, and tubulointerstitial fibrosis and hence microalbuminuria (24). Clinical studies confirmed that microalbuminuria is the first clinical sign of DN (18). Under normal conditions, charged protein particles are unable to pass through the barrier composed of glomerular basement membrane and podocytes. A trace amount of filtered protein will also be degraded and then reabsorbed in the proximal renal tubule. However, hyperglycemia-induced oxidative damage and subsequent inflammation may destroy the glomerular basement membrane, altering the membrane potential and apoptosis of podocytes, which results in the appearance of protein in the urine. Therefore, UAE is a useful marker of renal function in clinical tests (25). When compared with the DC group, supplementation of NRBE in diabetic rats can significantly decrease the UAE level, suggesting that NRBE may protect glomerulus from hyperglycemia-induced damage, possibly by its antioxidative capacity.

In addition, we found that oral administration of NRBE had no effect on the BUN or serum creatinine levels. As a traditional renal function marker, the serum level of BUN and creatinine could be affected by many factors, such as protein intake, degradation of lean body tissue, body dehydration, and stress (26). All streptozocin-induced groups, regardless of NRBE administration, showed no difference in BUN levels, which may be due to their similar food intake and weight loss. This indicates that all streptozocin-induced groups consumed a similar amount of protein, and their lean body tissues were degraded to a comparable level. Compared with the NC group, all streptozocin-induced groups showed higher food intake and less weight gain, and these results matched their higher BUN levels. These results also suggest that serum BUN and creatinine are insensitive markers in this animal model.

### NRBE enhanced the antioxidant status of diabetic rats

ROS is commonly used as indicator of oxidation level of animal tissue, and hyperglycemia raises the ROS level (27). GPx, an enzyme used to scavenge H_2_O_2_ and lipid peroxides in animal cells, is induced under oxidant conditions of animal tissues (28). Our results confirmed that hyperglycemia increased the concentration of ROS in the kidneys of DC rats, and NRBE treatments could reduce the ROS production and further reduce the induction of GPx. A large amount of GSH was oxidized into oxidized glutathione as a reducing agent for GPx, and the increase in GPx activity by high glucose simultaneously caused the GSH oxidation. The oxidation of GSH was decreased by NRBE treatment, indicating an improvement in intracellular antioxidant capacity through the GSH saving effect of NRBE in the diabetic groups. Hyperglycemia may decrease the activities of antioxidant enzymes by glycosylation of enzymes, and an animal study showed that the higher the oxidant pressure induced by diabetes, the higher the AGE values (29). When compared with the DC group, NRBE treatments decreased fasting blood glucose levels, possibly through increasing adiponectin secretion from adipose tissues (because adiponectin is a hypoglycemic hormone). It seems that the hypoglycemic and antioxidative effects of NRBE inhibit the chance of cross-linkage between glucose and protein, which contributes to a decrease in AGE formation in kidney tissues.

Our GPx results are in agreement with the findings of many other reports in that GPx activity was increased in kidneys under diabetic conditions, which is probably due to the self-defense mechanism of the body to raise GPx expression to prevent oxidant damage in the kidneys (30). SOD scavenging converted superoxide radicals into hydrogen peroxide, and then hydrogen peroxide was reduced by GPx to form water. Under hyperglycemic conditions, SOD activity was decreased in the DC group, but oral administration of NRBE in diabetic rats increased the expression of SOD in the kidneys. Increased SOD activity may decrease the production of superoxide radicals, hence leading to a decrease in GPx activity.

Many clinical studies and animal studies provide evidence that oxidative stress is one of the major causes of DN (31). ROS may destroy the glomerular basement membrane, altering membrane potential and apoptosis of podocytes, and resulting in glomerular enlargement, early albuminuria, and progressive glomerulosclerosis (32). Our results revealed that oral administration of NRBE decreased AGE and ROS production and, on the other side, increased GSH concentration and SOD activity in streptozocin-induced diabetic kidneys. However, a high dose of NRBE did not increase SOD activity compared with those of the DC group, which could imply that an overdose of NRBE might play two roles, as an antioxidant and oxidant agent, in animal tissues simultaneously (33). NRBE contained 6.30 mg gallic acid equivalent/g of total phenols and 12.03 mg rutin equivalent/g of total anthocyanin (data not shown), and therefore, it can provide powerful antioxidant protection. The –OH groups at the benzene ring perform as reducing agents, singlet oxygen quenchers, and metallic-ion chelators (34). Supplementation of NRBE can reduce oxidative pressure in the kidney via scavenging ROS by phenolic compounds of NRBE. Oral administration of medium-dose (200 mg/kg BW) NRBE showed the highest antioxidant activity in the kidneys of diabetic rats, and the same dose has also been proven to be the best dosage against DN.

### NRBE modulated renal inflammation of diabetic rats

Hyperglycemia-induced ROS and AGEs production may stimulate the secretion of proinflammatory cytokines, such as TNF-α and IL-6, in renal tissues. Renal TNF-α and IL-6 productions are highly related to the development of DN (35). An animal study showed that TNF-α expression increased in the glomerulus and proximal renal tubules in diabetic rats (36). TNF-α activation may induce apoptosis of renal cells, abnormal blood flow, and tubulointerstitial fibrosis, which result in glomerular damage (37). ROS promotes the production of TNF-α in renal mesangial cells, and AGEs also stimulate NO, ROS, TNF-α, and IL-1 secretion from macrophages, which cause inflammation and subsequent renal damage (5, 38–40).

IL-6 and MCP-1 also play an important role in inflammation. IL-6 increased in the renal tissues of streptozocin-induced diabetic rats and is related to kidney enlargement and albuminuria (41). The increase in IL-6 expression is related to kidney enlargement, an increase in albuminuria, glomerular basement membrane thickening, and mesangial expansion (42). MCP-1 is synthesized and secreted from renal tissues when stimulated by TNF-α. MCP-1 secretion worsens inflammatory tissues (43). AGEs may increase MCP-1 expression and secretion by activating the NF-kB-mediated signal transducing pathway in renal mesangial cells and the endothelium of renal tubules (44). CRP, an acute-phase protein of hepatic origin, rises in response to inflammation (45). The increased CRP level in the DC group indicated that streptozocin not only induced DM but also stimulated the inflammation of renal tissues. Researchers have shown a positive relationship between CRP and UAE in either type 1 or 2 diabetes (46–47).

In this study, we found that oral administration of low-dose NRBE (100 mg/kg BW) could decrease the renal IL-6 level, while a medium dose of NRBE (200 mg/kg BW) could reduce not only the IL-6 level but also TNF-α, serum CRP, and MCP-1 in diabetic rats. Medium-dose NRBE provides excellent antioxidant capacity and is an effective agent that ameliorates renal functions by lowing the expression of proinflammatory cytokines in diabetic rats.

### Active compound provided renal protection

Kampherol is an apigenin analog with good antioxidant capacity. Even kampherol was the major active phenolic compound in NRBE, however, its concentration was so little to be quantitated. NRBE contained high phenols and flavonoids, and we suspected that kampherol combined with other phenolic compounds exhibited antioxidant activities and provided renal protection effect. Kampherol could inhibit the proliferation of colon carcinoma cell lines, SW480 and Caco-2, by G2/M cell-cycle arrest (46). Research (48) found that the inhibitory activity toward colon cancer could be enhanced by combing 5–30 μM kampherol with 20 μM apigenin. Motar and AL-Hadad (49) found that kampherol was one of three active flavonoids in leaves, fruit and bark of *Casuarina cunninghamiana* L. Very few studies were focused on the bioactivities of kampherol, and the mechanism of kampherol on renal function is needed for further study.

## Conclusion

The appropriate amount of NRBE, 200 mg/kg BW in rats, will be recommended to prevent diabetic lesions by enhancing antioxidant status, decreasing AGEs, and inhibiting inflammatory reaction.
